# Construction Notes

**Published:** 1895-06-29

**Authors:** 


### CONSTRUCTION NOTES.

### Glasgow Samaritan Hospital.

The site of the new hospital consists of an isolated piece of ground extending to about three-fourths of an acre, and has a southern frontage towards Coplawhill Road. The building when completed will comprise two distinct blocks—the hospital and administrative—connected by corridors. The hospital block will consist of two floors, each containing four wards, accommodation being provided for 28 patients. The administrative block will consist of three floors. The mortuary, post-mortem rooms, wash-houses, laundry, &c., will be built apart from the two central blocks. The estimated cost of the building is £8,000. The architects are Messrs. M'Whannell and Rogerson, West Regent Street, Glasgow.

